# Atomic hydration potentials using a Monte Carlo Reference State (MCRS) for protein solvation modeling

**DOI:** 10.1186/1472-6807-7-19

**Published:** 2007-03-30

**Authors:** Sergei V Rakhmanov, Vsevolod J Makeev

**Affiliations:** 1 Institute of Genetics and Selection of Industrial Microorganisms, State Research Centre GosNIIgenetika, 1^st ^Dorozhny proezd, 1, Moscow, Russia; 2 Engelhardt Institute of Molecular Biology, Russian Academy of Sciences, Vavilova str. 32, Moscow, Russia

## Abstract

**Background:**

Accurate description of protein interaction with aqueous solvent is crucial for modeling of protein folding, protein-protein interaction, and drug design. Efforts to build a working description of solvation, both by continuous models and by molecular dynamics, yield controversial results. Specifically constructed knowledge-based potentials appear to be promising for accounting for the solvation at the molecular level, yet have not been used for this purpose.

**Results:**

We developed original knowledge-based potentials to study protein hydration at the level of atom contacts. The potentials were obtained using a new Monte Carlo reference state (MCRS), which simulates the expected probability density of atom-atom contacts via exhaustive sampling of structure space with random probes. Using the MCRS allowed us to calculate the expected atom contact densities with high resolution over a broad distance range including very short distances. Knowledge-based potentials for hydration of protein atoms of different types were obtained based on frequencies of their contacts at different distances with protein-bound water molecules, in a non-redundant training data base of 1776 proteins with known 3D structures. Protein hydration sites were predicted in a test set of 12 proteins with experimentally determined water locations. The MCRS greatly improves prediction of water locations over existing methods. In addition, the contribution of the energy of macromolecular solvation into total folding free energy was estimated, and tested in fold recognition experiments. The correct folds were preferred over all the misfolded decoys for the majority of proteins from the improved Rosetta decoy set based on the structure hydration energy alone.

**Conclusion:**

MCRS atomic hydration potentials provide a detailed distance-dependent description of hydropathies of individual protein atoms. This allows placement of water molecules on the surface of proteins and in protein interfaces with much higher precision. The potentials provide a means to estimate the total solvation energy for a protein structure, in many cases achieving a successful fold recognition. Possible applications of atomic hydration potentials to structure verification, protein folding and stability, and protein-protein interactions are discussed.

## Background

Most biochemical and biophysical processes take place in aqueous solutions. Interaction with water is the dominant force driving protein folding, providing approximately 90% of the total structure stability [1,2]. In many cases biological functions of macromolecules crucially depend on specific bound water molecules [3,4]. Water molecules bound in macromolecular interfaces significantly contribute to recognition of proteins by other proteins or DNA [5]. Successful ligand design also requires consideration of bound water molecules [6,7].

Protein solvation at the molecular level has been studied with different approaches varying from first principle modeling [1,8-10] to evolutionary considerations [11]. Here, we present a new approach to this important problem, which is based upon knowledge-based potentials (KBP) that proved to be efficient for modeling atomic interactions in biopolymers [12,13].

KBP are heuristic constructions [14] measuring the tendency of particular atoms and residues to form close contacts or to avoid each other in a macromolecular 3D structure. Statistical preferences of structure variables such as atom-atom contact distances in the conformation space are measured by log likelihood ratio [15]. These preferences can also be measured in energy units, when multiplied by a *kT *factor, the absolute temperature times Boltzmann constant [16]. The quasi-energy (the statistical preference) then takes the form of the Boltzmann equilibrium energy distribution:

E(d)=−kTln⁡fobs(d)fexp⁡(d)     (1)
 MathType@MTEF@5@5@+=feaafiart1ev1aaatCvAUfKttLearuWrP9MDH5MBPbIqV92AaeXatLxBI9gBaebbnrfifHhDYfgasaacH8akY=wiFfYdH8Gipec8Eeeu0xXdbba9frFj0=OqFfea0dXdd9vqai=hGuQ8kuc9pgc9s8qqaq=dirpe0xb9q8qiLsFr0=vr0=vr0dc8meaabaqaciaacaGaaeqabaqabeGadaaakeaacqWGfbqrcqGGOaakcqWGKbazcqGGPaqkcqGH9aqpcqGHsislcqWGRbWAcqWGubavcyGGSbaBcqGGUbGBdaWcaaqaaiabdAgaMnaaBaaaleaacqWGVbWBcqWGIbGycqWGZbWCaeqaaOGaeiikaGIaemizaqMaeiykaKcabaGaemOzay2aaSbaaSqaaiGbcwgaLjabcIha4jabcchaWbqabaGccqGGOaakcqWGKbazcqGGPaqkaaGaaCzcaiaaxMaadaqadaqaaiabigdaXaGaayjkaiaawMcaaaaa@4D4B@

Here *f*_*obs*_*(d) *is the observed frequency of contacts between atoms of two considered types at distance *d *in the database of macromolecular structures. Preferable atomic contact distances correspond to higher frequencies of atom contacts observed at this distance. It should be noted, that physical meaning of KBPs is not clearly defined, for instance, incorrect is a direct interpretation of (1) as Boltzmann distribution in some energy field [17]. In this study, atom types were defined as both residue- and atom-specific, e.g., a *CA_Val *atom type was assigned for the C_α_-atom of valine. The expected frequency of atom pair contacts *f*_*exp*_*(d) *is evaluated for a virtual state where the atoms do not interact, the so-called reference state. Calculation of the observed frequency *f*_*obs *_is relatively straightforward, although it depends on selection of the training data set, the binning procedure, and other technicalities. The principal difference between methods for construction of knowledge-based potentials lies in the definition of the reference state for calculation of *f*_*exp*_*(d) *[15,18-21].

Historically, KBPs were developed in parallel with construction of more complex and elaborate reference states. In the pioneering work of Tanaka and Scheraga [22], equations for complex formation at low concentrations were used as an implicit reference state. The quasi-chemical approximation reference state [18] employed an equilibrium mixture of unconnected residues to derive distance-independent inter-residue contact energies. Sippl [19] introduced distance-dependent potentials, and explicit accounting for chain connectivity. The "uniform density" reference state in [19] was based on the distribution of distances between all residue pairs of different types separated by **k **residues in the protein backbone averaged over a large training set of known structures. Subsequently, Samudrala and Moult [23] used conditional probability formalism, evaluating the probability for two atoms ***a ***and ***b ***to be found within a particular distance shell or bin in a correctly folded structure. Skolnick et al [20] introduced their composition-corrected reference state, with a direct dependence on the amino acid composition of individual structures from the training set.

In all the examples above, reference states were constructed via averaging of observed distances over pairs of residues of all possible types. Such atom type-averaged reference states tend to underestimate the interactions common to all types of atoms, such as atom repulsion at close distances or protein compaction due to solvent expulsion. However, inclusion of additional terms can compensate the effects of protein packing and compactness [24].

At small distances KBP are also plagued by small statistics of observed interactions. Therefore, the atom-atom contacts at distances closer than 2.0–3.0 angstrom are usually treated separately with the interaction potential at such distances set to some arbitrary prohibitive value. In the "ideal gas" reference state [21] which is not atom type averaged, the potential is also calculated separately for distances greater than 3.0 Å. This approach is based on uniformly distributed points in finite spheres with a complicated empirical dependence of a sphere radius based on the length of the protein backbone.

In this work, we present a novel method for construction of the reference state, which we have called Monte Carlo Reference State (MCRS). This method utilizes random 3D points in the structure volume as (by definition) non-interacting probes for calculation of the expected contact probability density distance distribution. These random probes are evenly distributed in the structure space regardless of the structure elements, thus providing a true zero interaction energy reference for atom-atom interaction. Monte Carlo methods have been long and successfully used in protein folding modeling ([22,25,26]) and atom interaction studies [27]. However, to our knowledge, no attempts have been made to use these methods for construction of the reference state.

Although MCRS technique can also be applied to produce knowledge-based potentials for interaction of protein atoms with each other, we used it to obtain KBP for hydration of protein atoms (atom hydration potentials, or AHP), based on the statistics of their contacts with structure-bound water molecules. Any atom locally distorts the interaction-free hydration density, obtained using MCRS. The direction and magnitude of this influence depends on the atom-water contact distance and on the hydropathy of the atoms of this type. Our aim in obtaining the AHP was to estimate the atomic hydropathy, quantitatively and in a distance dependent manner, for all types of atoms found in proteins.

Since water molecules are small and are not connected to the protein backbone, their location is primarily determined by intermolecular interactions of non-covalent nature. There are also comparatively many structure water molecules in the PDB database. Thus, the defined set of structural water contacts with different amino acids is relatively large, providing statistical power for our study. This suggests that KPBs for protein atom – water interaction can be obtained with greater detail and with less distortion than KPBs for residue-residue interaction used in protein folding.

## Results

### Water distribution in structural space

**Figure 1:**
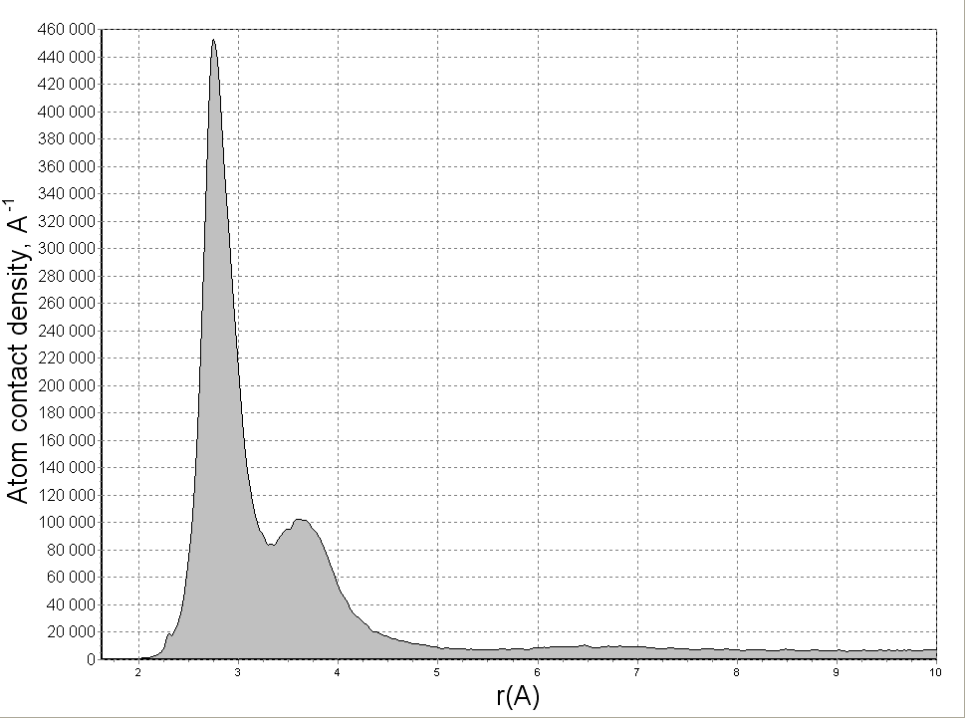
**Distribution of distances from a structure water molecule to the nearest protein atom**. Distribution of distances from a structure water molecule to the nearest protein atom for 319024 water oxygen atoms in 1776 structures. The two main peaks correspond to contacts of water with different oxygen and nitrogen atoms of proteins (the first peak at approximately 2.75 angstrom), and carbon atoms (the peak at ~3.7 angstrom).

**Figure 2:**
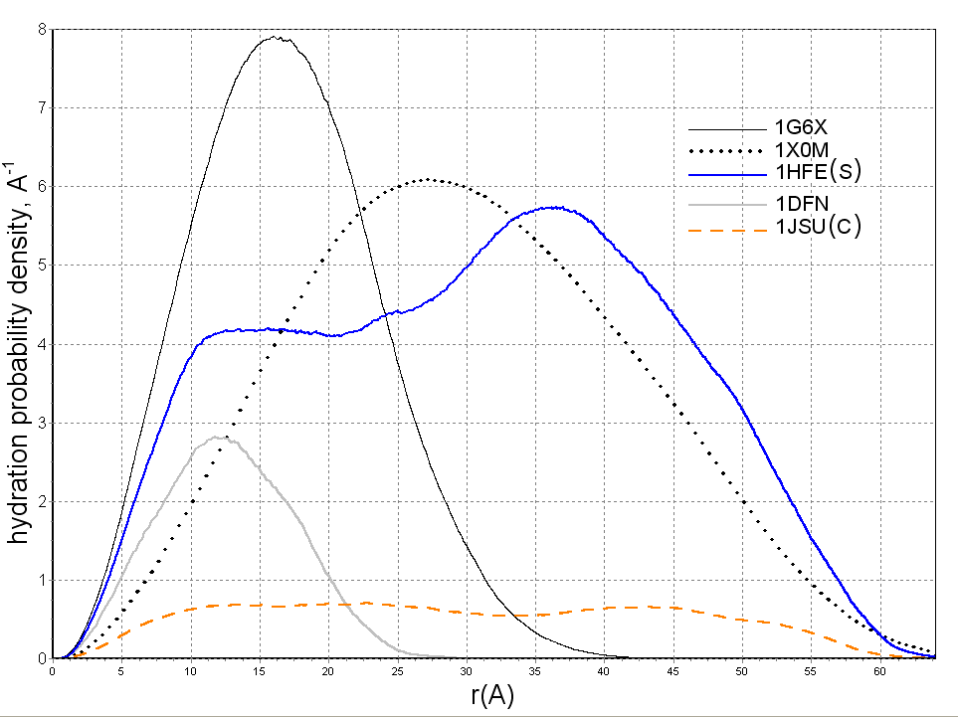
**Expected hydration contact densities, calculated using MCRS**. The expected hydration distance distributions of probability density calculated for five different protein structures. The area under each curve is equal to the number of waters in the structure.

Distribution of distances from a water oxygen to the nearest heavy (non-hydrogen) protein atom is shown in Figure 1. The first narrow maximum at approximately 2.75 Å corresponds to the solvent contacts with oxygen or nitrogen atoms, while the broader second peak is formed by water contacts with different protein carbon atoms. Figure 2 provides several examples of distributions of the averaged likelihood ratios for finding a water molecule at a given distance, from a random location within a structure, as calculated for five different structures. As it is evident from Figure 2, different proteins can have dramatically different expected distributions of distances from a random point within the structure volume to structure water molecules.

The probability curve depends substantially on the size and the shape of the protein molecule, and on water content and distribution. Bell-shaped hydration distance distribution curves are typical for roughly globular structures. In contrast, a bimodal distribution is obtained with the hourglass-shaped FE hydrogenase (1HFE) subunit S, and the low-value, long-range distribution of 1JSU, subunit C reflects an elongated 3D structure. Because of this variability, we believe that it is not possible to generate a reliable reference state by measuring atom-water distances in the aggregate for the entire training set, or by using common geometric parameters for some generalized average structure. In our approach, the distance distribution of atom-water contacts observed in a given structure is normalized by the expected distance distribution from a random point to water for that structure. This procedure automatically takes into account variations in structure size, shape, bound water content, and pattern of water distribution.

### Interaction of protein bound water molecules with each other

**Figure 3:**
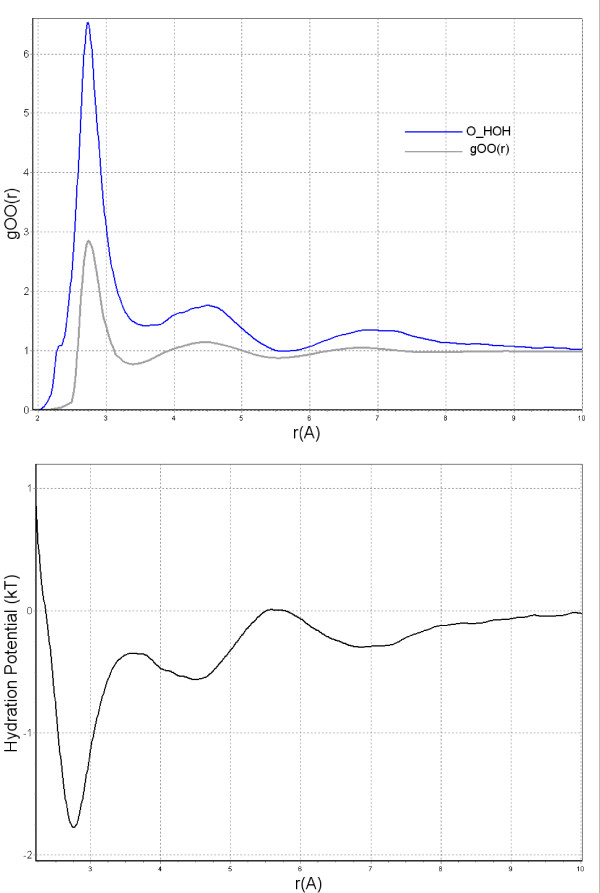
**Water-water empirical contact potential**. Normalized distribution of contact distances between water oxygen atoms in protein crystals (top) and the corresponding knowledge-based interaction potential (bottom), based on 54356851 contacts between 319024 crystallographic water oxygen atoms. The grey line gOO(*r*) in (*a*) shows oxygen-oxygen radial distribution function in the liquid water taken from [28].

The diagram in Figure 3 shows the likelihood ratio displaying the relative number of water oxygen atoms at a given distance from another water oxygen atom than from a random point in structure space. It reflects how a crystallographic structure water molecule influences other such molecules around itself. *F*_obs_/*F*_exp _values above 1 indicate preferred distances between water oxygen atoms and vice versa. In liquid water, a similar quantity called the *g*_*OO*_*(r) *factor or oxygen-oxygen radial distribution function is extracted from x-ray diffraction or neutron scattering measurements [28].

Although the structure of the liquid water and the distribution of the bound water molecules in protein structures are not directly related, the *F*_obs_/*F*_exp _ratio we obtained for protein crystal structure water agrees remarkably well with the *g*_*OO*_(**r**) plot for liquid water. The higher g_*OO*_(r) levels observed for protein crystals may be due to the excluded volume taken by protein atoms, which biases the expectation, and the fact that water molecules group around charged protein atoms, increasing the chances of observing one water molecule in the vicinity of another. The effective repulsion in the medium range in the *g*_*OO*_*(r) *for interacting structure water molecules is probably non-physical. In bulk water it appears to be a collective effect most likely related to formation of clusters of coordinated water molecules [29].

The three peaks at 2.75 Å, 4.5 Å and 7 Å reflect the layers of structured water molecules that are also present in water distance distribution around hydrophilic oxygen atoms within different protein groups as it can be seen from Figure 3(a).

### Interaction of bound water molecules with protein atoms of different types

**Figure 4:**
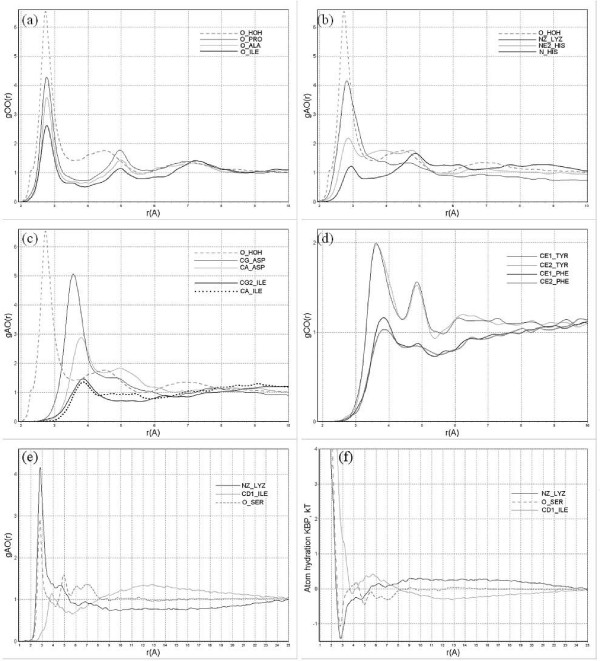
**Hydration potentials of several protein atoms**. Atom hydration likelihood ratios (a-e), and potentials (f) for atom contacts with explicit structural water, calculated for several protein atom types. (*a*) oxygen atoms of proline, alanine, isoleucine backbone groups; (*b*) nitrogen atoms: N_Z _of lysine, N_ε2 _of histidine, backbone N of histidine (*c*) carbon atoms: C_γ _of asparagine, C_α _of aspartate, C_γ2 _of isoleucine; (*d*) symmetric atom pairs: C_ε1 _and C_ε2 _of tyrosine and C_ε1 _and C_ε2 _of phenylalanine; (*e*) large scale behavior of distributions N_Z _atom of lysine, C_δ2 _of isoleucine, O of serine; (*f*) atomic hydration potentials, the same atoms as in (*e*). In (a), (b), and (c), the plot for water oxygen as in Figure 3, is given for reference.

Figure 4 gives several examples of likelihood ratios and KBP for protein atoms of different types. For reference, we also provide the hydration distance plot of structural water itself, which we have compared with water-water interaction in pure liquid in Figure 3.

Comparison of the profiles shown in Figure 4(a),(b) and 4(c) reveals that the location of the first maximum of preferable hydration distance is determined by the van der Waals radii of the water oxygen and the corresponding protein atom. The optimal hydration distance of carbon atoms (3.7 Å, Figure 4(c)) is considerably larger than that of nitrogen and oxygen atoms (2.8 Å, Figure 4(a) and 4(b)). For oxygen and nitrogen, the secondary maximum is also present at about 4.9 Å and 5 Å respectively, with an additional maximum at 7.2 Å. These additional maxima for oxygen are much sharper than the corresponding maxima in liquid water. This likely reflects stabilization of the structural water sites by the protein crystal, which causes better molecular resolution. In addition, the protein hydration shell itself may be more structured than the liquid water [30]. The first peak for oxygen and nitrogen atoms is generally narrower than for carbon atoms. This is probably related to the ability of hydrophilic oxygen and nitrogen atoms to form hydrogen bonds with water.

Atoms of similar types, but from different amino acids, may have different AHP, which reflects preferences for amino acid location in the protein hydrophobic core or at the surface, and the average solvent accessibility. For example, C_a _atom of isoleucine has a very different hydration distance plot than the C_a _atom of aspartate (Figure 4(c)). However, even the most hydrophobic residues and atoms, such as C_δ _of isoleucine or C_ε _of tyrosine (Fig. 4(d,e)), are occasionally exposed in solvent even in folded proteins, and thus exhibit the first peak at the van der Waals distance. One can see from Figure 4(b), that N_z _atom of lysine typically attracts significantly more water molecules than the backbone nitrogen of histidine. This happens because the N_z _of lysine has a substantial partial charge, and because this atom at the tip of a long side chain is more solvent-accessible. Figure 4(c) also shows how a partial charge at a carbon atom (C_γ _atom of aspartate) shifts the first hydration maximum to a smaller distance, most likely because the electrostatic forces of ion solvation attract water molecules. This effect is less pronounced for nitrogen or oxygen atoms.

The behavior of AHP at large distances also reflects the formation of hydrophobic core of the protein globule. For example, atoms within hydrophilic residues have a sharp peak at short distances, but a long basin in the range of 8–25 angstroms (Figure 4(e)). In contrast, atoms within hydrophobic residues have a much lower peak at short distances, and a very broad elevation in the 8–25Å range. This elevation is probably attributable to the typical distance from a residue buried in a protein hydrophobic core to the hydrated surface of the protein.

Residues with symmetric side chains and atoms, such as symmetric carbons in the aromatic rings of tyrosine or phenylalanine, provide a sort of a natural test for statistical atom contact potentials. These atoms are far enough from each other to have independent statistics of water contact distances, yet there is no physical reason why these chemically identical atoms in undistinguishable positions should have different potentials. Figure 4(d)) shows that indeed they are very similar. It also demonstrates the magnitude of the statistical error in the AHP obtained using MCRS.

### Prediction of hydration sites in protein structures

**Figure 5:**
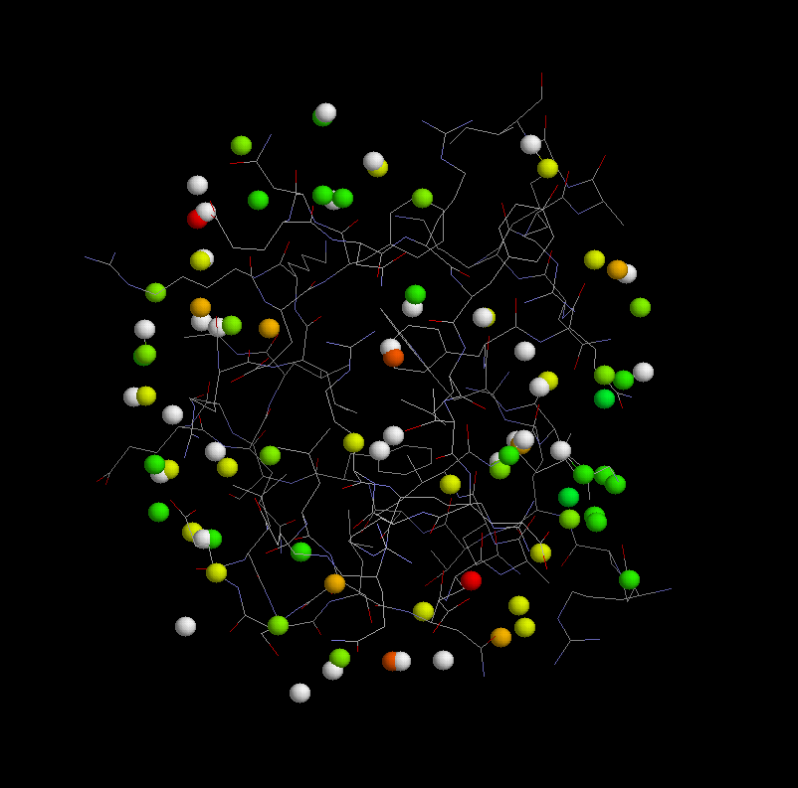
**Example of hydration sites prediction outcome**. An example of a hydration site prediction outcome. White spheres denote the experimental waters; colored spheres are the predicted hydration site location; the color of the predicted sites is according to the probability for observing a water molecule in the vicinity of the probe, red end of the spectrum corresponding to higher calculated hydration probability.

Using the AHP obtained by MCRS, we calculated putative bound water locations for several protein structures (see Methods for details). For some of the proteins studied, structural water locations were predicted earlier using Molecular Dynamics [31-33] or Monte Carlo simulation [34]. The results of our hydration site prediction experiments are summarized in Table 1. One example of such prediction is given in Figure 5.

As can be seen from the Table 1, the number of predicted hydration sites is equal to twice the number of the experimental water positions in that structure. In cases where the number of the lowest energy putative hydration sites equaled the number of reported waters for the structure, usually approximately 50 per cent of the experimental hydration sites were closely reproduced (with the distance from the structure water to the nearest predicted hydration site less than 1 Å). The percentage of correctly predicted structure waters was about 75–90% depending on the structure with the number of predicted hydration sites equal to twice of the number of real waters. This over-prediction is inevitable due to the fact that the number of structure-bound water molecules, reported by the structure depositors, may vary very much for similar structures. This problem is also addressed in 'the Discussion' section below. Routinely, it takes the number of putative hydration sites 3 to 10 times more than the number of reported waters, to reproduce most water locations [32]. A level of over-prediction as high as 40 times may be necessary to reproduce bound water well [34]. Predictions using AHP for the same structures have higher precision, a level of over-prediction reduced 2- to 5- fold, and are less demanding computationally.

### Decoy selectivity experiments

It is well-known [35] that hydrophobic interactions are decisive factors in protein folding. Thus, we tested the power of the AHP we had generated to select correct protein folds exclusively on the basis of hydration energy, using a decoy selectivity test. We used Decoys'R'Us database [36] used in [37] for evaluation of protein models by atomic solvation preference, and in [38,39] for fold recognition tests of an effective energy function using a Gaussian model for the solvation free energy.

To evaluate the differences in the hydration energy between the native and the decoy conformations, we introduced a geometric hydration shell (HS), defined as the 3D area in which water molecules can closely approach a heavy protein atom. Monte Carlo methods were used both to construct the HS, and to evaluate the number of water molecules within the HS (please see Methods section for the details).

The results of the decoy recognition tests are shown in Table 2, lines 1–23. For 23 out of the 23 structure sets, the native structures had the lowest average hydration shell energy normalized for the volume, as compared to the decoys. Relatively high ΔG differences between the native structures and the decoys indicate that hydration energy calculations using AHP allow one to clearly recognize the correct fold in 100% of the cases.

Inspired by the results of AHP application in this relatively simple decoy set, we tried a more demanding test. We used the improved Rosetta decoy set [40], which has an increased frequency of near native models and is considered to be a well-constructed decoy set obtained by large-scale comparative modeling [41]. Each of 41 native structures in the Rosetta set has between 1724 and 1900 decoys. Results are given in Table 2, lines 24–64. For 26 proteins, the native structure had the lowest hydration energy, often separated from those of all the decoys with a considerable energy gap amounting up to several standard deviations. Several of the structures tested could not be satisfactorily distinguished from their decoys using AHP. After close inspection we found that 'good' structures were mostly globular proteins with distinct hydrophobic cores and hydrophilic surfaces. Among unrecognized structures, e.g. 1ptq has a large hydrophobic patch at its surface and lacks a clearly defined hydrophobic core, whereas 1a32 has a loose packing with a number of hydrophobic groups accessible to the solvent. An interesting example is 1utg, which is a shell-shaped structure, with practically all hydrophobic residues at its concave side, thus providing a large hydrophobic momentum. The structure as it is given in the test set has all these hydrophobic residues accessible to the solvent, whereas in the native state, the protein forms a dimer, with its concave sides pressed against each other, forming a small hydrophobic cave. In our opinion, this test reveals the limits of applicability of our potentials in the field of the fold recognition, as mostly to soluble globular proteins.

## Discussion

### Comparison of atomic hydration potentials with other methods

Commonly used knowledge based approaches to modeling protein solvation are based on amino acid hydrophobicity or solvation preference, and solvation accessible surface area (SASA) calculations [42,43]. It has been noted that in protein-structure selections, all-atom based potentials perform better than residue-based potentials, and distance-dependent potentials better than distance-independent ones [[21], and the references therein]. In contrast to SASA-based methods, in AHP approach, no rigid atomic radii are set. Distance-dependent potentials allow considering spatial hydration shell rather than just the surface of the molecule. This may have an advantage in certain conformational scenarios, not distinguishable in terms of surface area.

A recent comparison [44] of five different protein solvation models, including a grid-based finite difference Poisson-Boltzmann procedure, demonstrated that the empirical atomic solvation model performed better than all the other models. At the same time [44] reports that protein design presents a particularly challenging test for implicit solvation models because it requires accurate estimates of the solvation contribution of individual residues. AHP introduce elaborate, distance-dependent representation of amphiphilic properties of individual protein atoms, which we believe may have important role in many practical applications.

First-principle physical models yield good approximations for water structure around solvated ions in solution [45], and have been applied to prediction of structural waters around protein globules [32,9]. The MCRS approach we have developed may have a number of advantages in addressing this problem. First, it allows calculation of the expected contact densities for any atom pair, to any desired precision, reducing the statistical error inevitable for the atom-type-averaged reference states. Second, it uses an individualized approach for each structure, taking into account protein shape, atom molar fractions and spatial distribution of atoms. Third, the MCRS scheme also generates detailed empirical short range atom interaction potentials. Figure 4(c) illustrates that AHP can characterize atomic interaction even at sub-van der Waalse distances, thus providing a unique capacity for quantitative description of atom contacts in this important distance range.

In principle, the MCRS allows one to obtain the expected atom-atom contact probability density distance distribution starting from zero distance, and for any individual macromolecular structure. The accuracy of the expected probability density estimates depends on the number of the random probes, which in turn is limited only by the computational power.

### Oxygen-oxygen distribution function in structural and bulk water

One particularly interesting result of this study is the oxygen-oxygen radial distribution function of the structural water. As Figure 3 shows, this function exhibits three peaks at similar distances to those observed in the oxygen-oxygen radial distribution function *g*_OO_(*r*) obtained experimentally for the liquid water [28]. The protein structure is likely to stabilize locations of bound water molecules [30], which agrees with the taller and narrower first peak in the distribution. However, water molecules in bulk liquid probably also form large clusters with quasi-stable structure [46,47]. The coincidence of main maxima in oxygen-oxygen distance distribution agrees with the hypotheses that the water around the protein basically retains its non-trivial structure found in the liquid [1,29,30].

The remarkable agreement demonstrated by Figure 3 not only indirectly justifies the accuracy of our technique, but also allows us to compare the packing of water molecules in liquid water and in protein crystals. Interestingly, while Molecular Dynamics simulation of pure liquid water reproduces well the experimental radial distribution function (and other physical properties) in water [48] and simple solutions [49], the same function of radial oxygen-oxygen contact frequency, obtained during MD simulation of solvated protein crystals, may have maxima locations and amplitudes different from those obtained for pure water [1].

### The quality of training data of structure water location and prediction of protein hydration sites

Evaluation of the quality of the prediction of structural waters reported in a PDB structure is not trivial. Favorable hydration locations can produce a diffuse electron density and be excluded from the structure [1]. Thus, only a fraction of strongly bound water molecules in protein crystals is usually reported. We observed that the number of waters detected for similar structures may vary by two orders of magnitude (for instance, compare two structures of the same HIV-1 protease-inhibitor complexes: 2BPY with 115 reported water molecules, and 1HVK with just one). Second, a large fraction of the structural waters reported for X-ray structures are probably stabilized not only by the protein molecule, but by the crystal unit cell. Indeed, [50] reported that only 17 structural water molecules were found at the same sites in nine crystals of ribonuclease A resolved from five different space groups and containing from 88 to 188 water molecules. Similar results have been published for other proteins [51,52] and discussed in [53]. The extremely variable water content reported for different proteins most likely reflects the fact that not all suitable sites are occupied, and this obviously hinders verification of location of hydration sites. Nevertheless, we believe that despite a considerable noise in the input data, our procedure still affords to capture the essential aspects of the interaction potentials at the atomic level provided enough statistical data, as illustrated by Figure 3(d), and allows us to provide a valuable prediction of putative structural water sites.

### KBP for analysis of known structures

**Figure 6:**
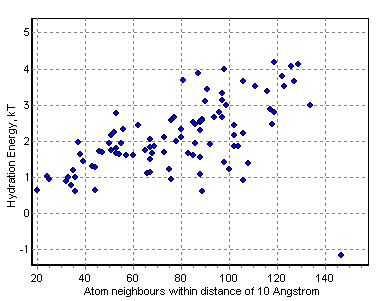
**Water in a protein hydrophobic cavity hydration energy estimation**. Hydration energies (kcal/mol) *vs*. atom contacts number plot for some hydration sites of 1TSF, calculated using AHP. Note the rightmost water below at the bottom of the diagram (circled).

The currently weak definition of experimentally obtained bound water molecules suggests the value of KBP for verification of reported structural waters. The presence of water in protein hydrophobic cavities has been a source of controversy. Hydrophobic cores in proteins sometimes have cavities large enough to accommodate water molecules. Water in such cavities is often missed in X-ray, but detectable by NMR [54,55]. The expected average water occupancy for a cavity is determined by a balance of the entropy factor driving water into the cavity, proportional to its volume, and the enthalpy of water contacts with the protein atoms lining the cavity. Our calculations using AHP show that the free energy change for the transfer of a water molecule into compact all-hydrophobic cavities from outside of a protein is very unfavorable. This indicates that extremely hydrophobic small cavities probably lack water molecules most of the time, so that the chances for observing a water molecule are very low at any particular position within the cavity. Our analysis agrees with the observations that disordered water molecules may be present, at least transiently, in large hydrophobic cavities in proteins [56]. Nevertheless, we observe that some published protein structures have one or more water molecule, placed at a highly energetically unfavorable location, usually into a closely packed hydrophobic environment. Figure 6 illustrates this situation. Possibly, the source of the corresponding electron density should be inspected and the structure might be ameliorated.

Use of the AHP allows calculation of the probability that a water molecule will occupy a certain site, or, equivalently, the proportion of time that a molecule of water is present within a certain volume in the protein environment. Moreover, individual inputs from atoms and residues into, for instance, transmembrane channel permeability or a fold solvational stability can be estimated.

### MCRS KBP at short and large distances

AHP at short distances offer a high resolution water assignment. At large distances (see Figure 4(f)), the potentials allow applying them to model cooperative processes like protein folding. For decoy recognition and similar applications, the behavior of KBP at large distances (Figure 4e,f) is important. These distances are comparable with the average radius of a protein globule, thus global structures as a hydrophobic core may be discerned. At these distances, the knowledge-based potentials work as statistical models, rather than provide a description of pairwise physical interactions.

### Possible applications of the KBP for protein folding and interaction

Solvation term is the single most important factor, usually providing about 90% of the total structure stability [57]. Models for protein folding were even proposed which included only the hydrophobic interactions of amino acids [58].

In the decoy recognition tests we evaluated the change in the hydration energy of the entire structure that accompanies changes in protein conformation. These tests ignored any intra-molecular interaction, i.e. between different protein atoms and groups. As such, evaluation of hydration energy with AHP can be complementary to other potential sets and atomic force fields in estimation of structure stability, by adding an explicit accounting of the solvation forces.

Accounting for hydration changes at the atomic level is crucial for interaction of macromolecules. Even indirect account for water mediated interactions improve significantly the quality of analysis of interaction of supersecondary structural elements in protein folding, which probably is true also for protein binding [59].

AHP also allow calculating the desolvation energies accompanying mutual occlusion of different parts of hydration shells during protein-protein or protein-DNA interaction and ranking of different binding variants with regard to desolvation.

## Conclusion

We present a novel method for constructing the reference state for knowledge-based inter-atomic potentials, based on a Monte Carlo technique (MCRS). Using it, we have developed original potentials for protein atom hydration. The new potentials provide a detailed quantitative description of atom hydropathies in a distance-dependent manner. Using the new potentials allows calculated placement of individual water molecules in a protein environment, and estimation of the (de)solvation energy changes accompanying protein conformational changes and interaction.

## Methods

### Training database

The training database contained 1776 3D structure of proteins and protein subunits, with sequence identity between any pair of proteins less than 25% [60]. For each structure, all explicit water molecules located within 4.5Å from any structural atom were collected. Figure 1 illustrates this selection of the cutoff distance. Coordinates of only the electron-dense oxygen atoms were used for water molecules.

Crystallographically symmetrical positions were calculated for all water molecules, using the unit cell data and symmetry transformation operators provided in the PDB file. If such transformation yielded a symmetrical location, which was at sterically acceptable but closer distance to any of the protein atoms, the new position was adopted for the corresponding water molecule.

Distances from each protein atom to each water molecule were calculated and stored for each atom type. Our definition of atom type takes into account the specific amino acid context and the elemental identity, e.g., a C_α_-atom of valine is different from C_α_-atom of leucine, different from C_β_-atom of leucine. No effort was made to group atom types on the basis of their hydropathy properties, or to cluster them depending on their determined hydration potentials.

### Constructing the Monte Carlo Reference State

For each structure in the training set, probes with random coordinates were generated in the rectangular box encompassing the structure plus 2.5 Å in every direction. The number of probes was sufficient to produce a stable atom-probe distance distribution, and typically was 100-fold greater than the number of atoms in the structure. We discarded isolated probes separated by more than 2.5 Å from any heavy protein atom. For each of the remaining probes, a distance distribution to all the explicit water molecules was computed and stored. This quantity was aggregated for all probes, and divided by the number of probes, to obtain the structure-dependent empirical distribution of the probability to find a water molecule at the given distance from a random point in the structure volume. From this empirical distribution the smoothed probability distribution, or hydration probability density distance distribution for structure-specific MCRS was calculated using Parzen kernels [61]. This approach is known to give a consistent estimation of probability density distribution and has been successfully used in a study of statistical preferences in protein structures [62]. Several examples of such distributions are given in Figure 2.

### Calculating knowledge-based atom hydration potentials

For each structure in the training set and for every atom type we calculated distribution of atom-water contact distances. This was done in a manner similar a MCRS calculation, but with structural atoms of a particular type used instead of random probes. For each test atom type and for each structure, the observed atom-water distance distribution was divided by the expected atom-water distance distribution derived for that structure using MCRS. The resulting likelihood ratios were summed for all structures in the training set. This aggregate was divided by the overall number of the test atoms in the training set and smoothed using Parzen kernels [61]. Examples of such empirical normalized distance distributions are given in Figure 4(a–e) and are also provided in the Additional files [see Additional file 1]. A logarithm of this value multiplied by minus *kT *factor produces the KBP for hydration of the test atom type (Figure 4(f)). The distance-dependent hydration potentials thus obtained quantitatively describe atomic hydropathies of all atom types found in proteins, DNA and some hetero groups, such as ions, hem groups etc. Characteristic features of the potentials were robust in spite of variations in the training database composition; to establish this point, the training protein structure dataset was split into various subsets, and the potentials were recalculated for each subset separately.

### Prediction of location of structural water molecules

For this test, we selected 16 diverse protein structures based on criteria including the published computational predictions of water locations; a small to medium structure size; overall structure quality, and the number of structure water molecules. Water molecules were pre-filtered to remove those with any heavy protein atom closer than 2.0Å, or with no protein atoms within 4.5Å; such waters in the 16 structures were few, or none. Virtual probes were placed at the nodes of a 0.2 Å cubic grid encompassing complete structure volume. Nodes with any heavy protein atom closer than 1.8Å, or with no protein atoms within 4.5Å, were discarded. For all the remaining probes, the local hydration energy was estimated as the sum of hydration potentials contributed by all protein atoms within 15 Å. The grid step of 0.2 Å ensured that the estimated aggregate hydration energy was comparable at adjacent grid nodes.

In the next step, the probe with the hydration energy minimum for the entire structure was selected and all nodes within 2.6 Å around it were removed. This was repeated for the next best minimal energy probe, and continued until only the probes located at local minima of hydration energy and not closer to each other than 2.6 Å remained. The minima positions were further corrected via iterative steepest descent energy minimization algorithm. This rapidly converged to very small displacements of probes per one step. Inter-probe water-water interactions (Figure 3) were added during this stage. The resulting set of predicted structural water positions proved robust to variations in the grid step and to the initial placement of probes. For instance, a very close set of predicted structure water locations was obtained when a comparable number of probes were initially located at random points in the structure space.

The number of predicted hydration sites (lowest hydration energy probes) for each of the 16 structures (Table 1) was set to twice the number of the experimentally determined structural water molecules for the corresponding structure (please see Discussion section for the explanation of the increased number of probes in the prediction). The positions of predicted probes were compared to those of the experimental structural water molecules.

The prediction quality was assessed using a Z-score calculated as:

z = (RMSD_random _- RMSD_predicted_)/s_random_,

where RMSD_predicted _is the root mean square deviation of predicted hydration sites from the crystal structure water locations, and RMSD_random _and s_random _are respectively the mean similar quantity calculated for the same number of random probes in the structure volume, and its standard deviation. The latter is obtained in a computational experiment with the same steric limitations imposed on the probe – protein atom distances as used during prediction.

### Decoy selectivity experiments

For the decoy selectivity tests, a Decoys'R'Us 'hg_structal misfold' [36] decoy set was used, for Table 2, lines 1–23, and, and the improved Rosetta set [39] for Table 2, lines 24–64.

Randomly placed probes were generated in a rectangular box containing the structure with an extra 4.5Å added from all sides. Probes with any heavy protein atom closer than 2.5Å or with no protein atoms within 4.5Å were discarded. The remaining probes were considered as belonging to the protein hydration shell (HS). For simplicity, the volume of this shell was estimated via the ratio of the number of probes assigned to the HS to the total number of probes created in the box. To discriminate between the native fold and the decoys, per-volume average hydration energy of probes in the hydration shell was selected as a criterion. Table 2 summarizes the results.

## Authors' contributions

SR and VM both contributed to the development of theoretical basis and writing of the manuscript. SR conceived the study and carried out its programming implementation. Both authors read and approved the final manuscript.

## Supplementary Material

Additional file 1 Atomic Hydration Potentials. The distributions provided in the (archived with WinRar) graphical form (bitmap file format, .bmp) are normalized likelihood ratios for protein atom contacts with bound structure water molecules. Atomic hydration potentials in digitalized form and using it software tools for macromolecular solvation analysis can be found at the web page of the Laboratory for Bioinformatics at the Institute for Genetics and Selection of Industrial Microorganisms, State Research Center GosNIIGenetika. Click here for file

## Figures and Tables

**Table 1:** Prediction of hydration sites in 16 structures

##	Protein	Number of Amino Acids	Structural waters (experimental)	Hydration sites predicted	Water prediction average RMSD	**Z-score**
1	1AJJ	37	30	60	1.02	**6.97**
2	1ECA	136	94	188	1.72	**4.72**
3	1ENH	54	33	66	0.87	**5.36**
4	1FFO	202	122	244	0.91	**9.18**
5	1IRD	287	325	650	1.44	**2.69**
6	1KR7	110	106	212	0.86	**9.60**
7	1MAA	540	187	374	1.32	**5.41**
8	1MOF	53	39	78	0.90	**7.34**
9	1SBX	103	79	158	0.86	**10.41**
10	1UOY	64	126	252	1.03	**7.73**
11	4MT2	62	69	138	1.35	**4.48**
12	5PTI	58	63	126	1.07	**8.53**
13	1IFC	131	206	412	1.49	**6.15**
14	2PTH	193	175	350	1.68	**5.91**
15	3EBX	62	82	164	1.58	**4.52**
**16**	**4PTI**	**58**	**57**	**114**	**1.29**	**7.28**

Prediction of hydration sites in 16 structures (PDB IDs are indicated). Twice the number of reported experimental water molecules was predicted as hydration locations, to achieve the given average RMSD between the predicted and the actual positions of water molecules.

**Table 2:** Decoy recognition based on total structure hydration energy estimates

#	Structure ID	Hydration Shell Energy Average, kT		Native to Decoy ΔG, kT\Z-Score	**Enrichment, %**
				
		Native	Decoy		
1	5RXN	-7.22	-6.13	1.09	**100**
2	1REI	-15.21	-11.98	3.23	**100**
3	2CRO	-6.42	-5.57	0.85	**100**
4	2SSI	-8.77	-7.45	1.32	**100**
5	2PAZ	-7.34	-5.59	1.75	**100**
6	1LH1	-13.82	-9.63	4.59	**100**
7	2ILB	-6.83	-5.92	0.91	**100**
8	5PAD	-12.95	-11.41	1.54	**100**
9	1P2P	-13.24	-10.51	2.73	**100**
10	1RHD	-11.98	-10.62	1.36	**100**
11	2CI2	-8.65	-7.46	1.19	**100**
12	2B5C	-7.36	-5.68	1.68	**100**
13	2TMN	-10.160	-9.10	1.06	**100**
14	2CYP	-12.208	-9.33	2.88	**100**
15	2bp2	-9.635	-7.90	1.73	**100**
16	1cbh	-10.17	-8.65	1.52	**100**
17	1fdx	-8.12	-7.09	1.03	**100**
18	1hip	-6.14	-5.40	0.74	**100**
19	1ppt	-11.01	-6.98	4.03	**100**
20	1rn3	-12.09	-10.50	1.59	**100**
21	1sn3	-8.89	-7.38	1.51	**100**
22	2cdv	-10.27	-8.84	1.43	**100**
23	2ts1	-11.77	-9.75	2.02	**100**
24	1res	-11.30	-9.63	5.10	**100**
25	1ptq	-10.21	-9.70	0.68	**74**
26	1uxd	-10.04	-8.27	5.08	**100**
27	2pdd	-8.68	-8.73	0.04	**51**
28	1uba	-9.01	-8.28	1.24	**88**
29	1gab	-7.71	-5.98	3.25	**100**
30	1vif	-10.05	-8.70	2.37	**98.5**
31	1bq9	-10.22	-8.01	3.55	**100**
32	5pti	-11.12	-8.31	4.70	**100**
33	1aa3	-9.94	-7.76	3.78	**100**
34	1bw6	-7.962	-7.564	0.80	**79**
35	1orc	-8.944	-9.693	-1.04	**15**
36	1am3	-10.857	-9.126	3.67	**100**
37	1pgx	-10.211	-8.262	3.40	**100**
38	1tif	-10.283	-8.416	3.33	**100**
39	1msi	-12.712	-10.401	3.69	**100**
40	2ptl	-10.982	-7.708	6.89	**100**
41	1r69	-10.316	-7.670	4.69	**100**
42	1tuc	-10.564	-8.614	2.28	**100**
43	1dol	-10.890	-8.611	3.68	**100**
44	1utg	-7.998	-7.792	0.42	**67**
45	1csp	-11.524	-8.227	4.30	**100**
46	1a32	-9.844	-10.077	-0.50	**28**
47	2ezh	-11.496	-9.431	3.74	**100**
48	1nre	-8.198	-7.288	1.92	**97**
49	1sro	-11.319	-9.443	2.85	**99.5**
50	2fow	-9.442	-7.466	5.24	**100**
51	1ail	-10.957	-10.734	0.38	**63**
52	1ctf	-8.758	-7.123	3.30	**100**
53	1lfb	-9.387	-7.908	3.18	**100**
54	1nkl	-11.289	-10.048	2.21	**99**
55	1pou	-11.890	-9.495	4.40	**100**
56	1mzm	-12.126	-9.837	4.93	**100**
57	1afi	-10.691	-9.655	2.28	**99.5**
58	5icb	-9.006	-6.895	5.80	**100**
59	1kjs	-10.521	-9.063	2.90	**100**
60	1hyp	-14.662	-12.513	2.63	**98.5**
61	1cc5	-10.227	-9.895	0.64	**77**
62	1vcc	-13.279	-11.533	2.71	**100**
63	2fxb	-11.126	-8.699	4.37	**100**
**64**	**1cei**	**-9.810**	**-9.589**	**0.43**	**65**

The average hydration energy of the structure hydration shell, calculated with AHP, was used as a discriminating criterion for the fold recognition test. Column five gives the estimated free energy difference between the native and the decoy structures (lines 1–23), or a Z-score in case of the multiple decoys (lines 24–64). The last column, 'Enrichment', gives the percentage of the decoys with the estimated free energy higher (not as good) than the native structure. Decoys'R'Us database hg_structal misfold decoy set [36] was used for this test, lines 1 to 23, and the improved Rosetta set (lines 24 to 64), made by Tsai et al. [39].
